# Clinical Features of Families with a Novel Pathogenic Mutation in Sepiapterin Reductase

**DOI:** 10.3390/ijms26073056

**Published:** 2025-03-27

**Authors:** Feda E. Mohamed, Lara Alzyoud, Mohammad A. Ghattas, Mohammed Tabouni, André Fienemann, Joanne Trinh, Ibrahim Baydoun, Praseetha Kizhakkedath, Hiba Alblooshi, Qudsia Shaukat, Rim Amouri, Matthew J. Farrer, Samia Ben Sassi, Fatma Al-Jasmi

**Affiliations:** 1 Department of Genetics and Genomics, College of Medicine and Health Sciences, United Arab Emirates University, Al Ain 15551, United Arab Emirates; fedah.h@uaeu.ac.ae (F.E.M.);; 2 ASPIRE Precision Medicine Research Institute Abu Dhabi, United Arab Emirates University, Abu Dhabi 15551, United Arab Emirates; 3 College of Pharmacy, Al Ain University, Abu Dhabi 64141, United Arab Emirates; 4 AAU Health and Biomedical Research Center, Al Ain University, Abu Dhabi 64141, United Arab Emirates; 5 Institute of Neurogenetics, University of Lübeck, 23538 Lübeck, Germany; 6 Department of Pediatrics, Tawam Hospital, Al Ain 15258, United Arab Emirates; 7 Neurology’s Department, Mongi Ben Hmida National Institute of Neurology, Tunis 1007, Tunisia; 8 Department of Neurology, University of Florida, Gainesville, FL 32611, USA; m.farrer@ufl.edu

**Keywords:** developmental delay, ataxia, parkinsonism, cognitive delay, neurotransmitter disorders

## Abstract

Sepiapterin Reductase Deficiency (SRD) is a rare inherited neurometabolic disorder caused by variants in the *SPR* gene, which may lead to developmental delays, psychomotor retardation, and cognitive impairments. Two consanguineous North African and Middle Eastern families are reported with multiple affected individuals presenting with developmental delay, ataxia, hypotonia, fatigue, and ptosis, or parkinsonism and cognitive impairment. Exome sequencing revealed a novel homozygous *SPR* c.560A>G (p.Glu187Gly) mutation that segregates with disease. According to molecular dynamics analysis, the substitution is predicted to compromise structural integrity, likely affecting ligand binding and catalytic activity. Elevated cerebrospinal fluid sepiapterin and biopterin levels, along with low neurotransmitter levels, were concordant with a genetic diagnosis of SRD and the reclassification of this variant as pathogenic. SRD patients manifest a broad constellation of symptoms, albeit well-managed using low-dose L-dopa/carbidopa. This study highlights the value of genetic testing in expediting early diagnosis and intervention to mitigate the onset of this disorder.

## 1. Introduction

Sepiapterin Reductase Deficiency (SRD; MIM: 612716) is an autosomal recessive disorder characterized by severely low neurotransmitter production of dopamine and serotonin, resulting from deficits in tetrahydrobiopterin (BH4) synthesis [1]. Bonafé et al. (2001) first reported patients with a constellation of features, including progressive psychomotor retardation, dystonia, ataxia, spasticity, growth retardation, and cognitive dysfunction [2]. The disease has primarily been attributed to pathogenic mutations in the *SPR* gene [3], which encodes sepiapterin reductase (SR; EC 1.1.1.153), an enzyme that plays an essential role in the biosynthesis of BH4 from sepiapterin, utilizing NADP(H) as a cofactor. To date, 36 disease-causing mutations are listed in the Human Gene Mutation Database (HGMD), including 23 missense/nonsense, three splicing sites, three regulatory substitutions, five indels, and one gross deletion [4]. The rarity of this syndrome, along with the number of pathogenic mutations and their presentations, make diagnosis and treatment challenging. Diagnosis of SRD is generally based on deficits in neurotransmitter metabolites in cerebrospinal fluid (CSF), despite normal levels of phenylalanine in plasma. This is confirmed through measurement of SR activity in patient fibroblasts and/or *SPR* mutational analysis [5]. Most patients benefit from precursors of catecholamine synthesis, including the administration of 5-hydroxytryptophan and L-dopa in combination with carbidopa [6]. Here, we describe the presentation and progression of symptoms in two consanguineous families of Palestinian and Tunisian origin with recessively inherited SRD resulting from a novel homozygous *SPR* mutation.

## 2. Results

### 2.1. Family History and Clinical Presentations

#### 2.1.1. Family 1

**Figure 1:**
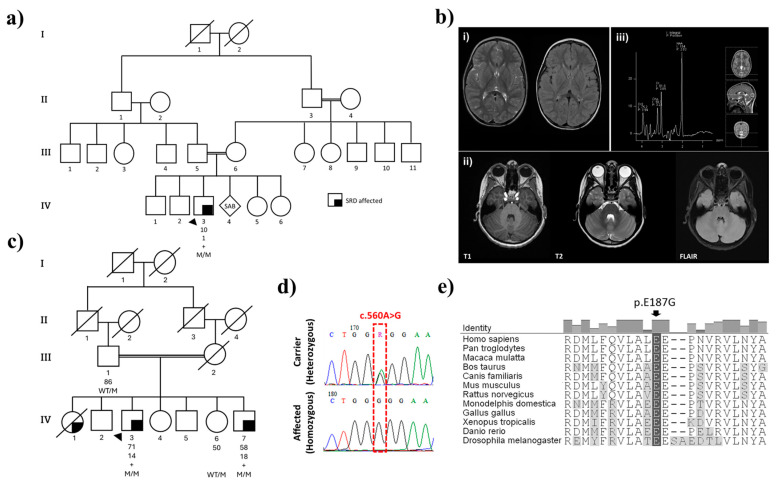
Clinical and molecular characterization of SRD-affected families (**a**) Pedigree of the consanguineous Palestinian family. (**b**) Magnetic resonance images of Pal_IV-3 brain showing: (**i**) a small hyperintense focus on the left periventricular region; (**ii**) a normal cerebellum; and (**iii**) normal brain magnetic resonance spectroscopy. (**c**) Pedigree structure of the consanguineous Tunisian family. Lower-right filled symbols indicate affected individuals with SRD. The black arrow denotes the proband. Age and age at onset are written underneath the symbols. M refers to the SPR c.506A>G (Glu187Gly) mutant allele, WT stands for wild-type, and + denotes members who underwent exome sequencing. (**d**) Sanger sequencing chromatograms illustrate SPR WT/M and M/M genotypes (**e**) Cross-species conservation of SPR showing protein orthologs aligned with ClustalW. The amino acid position of Glu187 (p.E187G) is boxed in black, with residues that differ from the human sequence in gray. RefSeq accession numbers: Homo sapiens NP_003115; Pan troglodytes JAA35602; Macaca mulatta NP_001247478; Bos taurus AAI18300; Canis lupus familiaris XP_540234; Mus musculus NP_035597; Rattus norvegicus NP_062054; Monodelphis domestica XP_001362257; Gallus gallus XP_423038; Xenopus tropicalis NP_001120067; Danio rerio ABC68460; Drosophila melanogaster NP_727265.

The proband (Pal_IV-3) is a 10-year-old male born to a consanguineous first-cousin marriage originally from Palestine (Figure 1a). The family consists of four healthy siblings with an otherwise negative family history. The affected proband had an unremarkable perinatal history but was initially evaluated for hypotonia, developmental delay, ataxia, and fatigue. He was born at term through normal vaginal delivery with no history of asphyxia or hypoxic-ischemic encephalopathy. However, he experienced a gross motor delay, supporting his own head and neck at nine months, rolling over at one year, and walking at three years old. He exhibited an inability to balance and walked with clumsy, staggering movements, demonstrating a wide-based gait. As an infant he frequently fell, couldn’t run well, and needed support going up and down stairs. Additionally, he was unable to ride a tricycle. His motor performance and activity levels varied throughout the day, with noticeable diurnal fluctuations, where he exhibited more pronounced difficulties during the later hours. The proband also displayed fine motor difficulties, evidenced by intention tremors while writing his name, drawing simple shapes, and struggling with button management. Tying his shoelaces has proven to be a challenging task. His speech development was slow, characterized by pronunciation difficulties and immature speech. He faced learning difficulties, particularly in following complex commands. Currently, the proband is receiving special education to address his learning disabilities. His physical examination showed mild hypotonia, good strength in all four limbs, normal deep tendon reflexes (DTR) in the upper limbs, and brisk DTR in the lower limbs, with an ataxic gait and a positive Romberg sign. He had coarse intention tremors, with no past pointing nor nystagmus. He received physiotherapy and started using a customized ankle and foot splint.

Brain magnetic resonance imaging (MRI) conducted in the first year of life showed a lack of white matter with the occipital horns of the lateral ventricles, with tiny foci of nonspecific high signal intensity in the periventricular white matter on the T2-Weighted Imaging (T2WI). Otherwise, brain myelination was within normal ranges for the patient’s age. There was no evidence of atrophy or hydrocephalus. The suprasellar cistern, brainstem, and cerebellum were normal. A nerve conduction study (NCS) performed at three years of age was normal, while the electromyography (EMG) showed signs of chronic and mildly active denervation. A brain MRI was repeated at the age of eight, upon referral to Tawam Hospital, showing similar results (Figure 1b). MRI of the thoracic spine showed normal spinal cord thickness with no atrophic changes observed. The spinal canal was adequate, with no disc herniation or protrusion. The lumbar spine MRI was unremarkable and there was no obvious pathology of the cervico-thoracic spine. Brain magnetic resonance spectroscopy (MRS) results were normal, showing high N-acetyl aspartate peaks, albeit within normal limits. The creatinine/choline ratio was also typical, without excessive choline peaks.

The proband (Pal_IV-3) cerebrospinal fluid also showed normal parameters for glucose (3.8 mmol/L [normal reference: 2.5–4.4 mmol/L]), protein (0.29 g/L [normal reference: 0.47–0.81 g/L]), and RBCs (<1 cell/mm^3^), and bacterial cultures came back negative. However, neurotransmitter measures showed low 5-hydroxyindoleacetic acid and homovanillic acid, suggestive of dopa-responsive related dystonia (Table 1). Further tests revealed elevated levels of sepiapterin and dihydrobiopterin metabolites, consistent with genetic analysis and a clinical phenotype of SRD. The patient was initiated on levodopa/carbidopa at a dose of 0.5 mg/kg/day of levodopa, co-administered with carbidopa in a 4:1 ratio. Additionally, 5-hydroxytryptophan (5-HTP) was prescribed at a dose of 0.5 mg/kg/day. Both medications were administered twice daily in divided doses. However, the patient was unable to swallow the 5-HTP capsule, and the syrup formulation was unavailable; thus, he continued with L-dopa/carbidopa only. His mother reported positive changes with medication, including improved attention and school performance, lessened ptosis, increased activity, heightened alertness, the ability to stay awake until midnight, and an improvement in his diurnal fluctuation. Before starting the medication, the patient would typically feel fatigued around 5 p.m., accompanied by ptosis, and would need to sleep to alleviate symptoms. The dosage is maintained at 2 mg/kg/day due to its benefits, despite side effects, including involuntary facial and eye twitching, abdominal pain, and enuresis.

#### 2.1.2. Family 2

A Tunisian family was referred with three affected siblings. The pedigree showed third-degree consanguinity and an autosomal recessive pattern of disease inheritance (first cousins; Figure 1c). Detailed clinical information is available for two brothers who were reported with atypical juvenile-onset parkinsonism at age 14 (Tun_IV-3) and 18 (Tun_IV-7). The proband, Tun_IV-3, initially presented at age 9 with paroxysmal generalized weakness that caused frequent falls, with diurnal fluctuations and sleep benefit. He had tremor in both hands, dysphagia, and dysarthria. CSF showed normal parameters of glucose (3.9 mmol/L), protein (0.24 g/L), and RBCs (2 cells/mm^3^), and cupric tests were normal.

Further examination at age 32 revealed an ataxic gait, parkinsonism with bradykinesia and rigidity, and mild tremor in both upper limbs. He was treated with L-dopa, which led to a dramatic improvement. Since age 60, he has developed dyskinesia, albeit without motor fluctuations. However, he has mild cognitive impairment, with a frontal dysexecutive syndrome (Mini-Mental State Examination (MMSE) = 25/30; Frontal Assessment Battery (FAB) = 6/18), but no psychiatric symptoms. He has no dysautonomic symptoms or sleep disorders. At his last examination at age 71, off medication, he had dysarthria, severe generalized dyskinesias, mild parkinsonism with bradykinesia and rigidity in both upper limbs, and mild bilateral postural tremor in both hands. His current treatment regimen consists of Modopar (375 mg/day) and trihexyphenidyl (7.5 mg/day). While he experiences occasional slowness in daily tasks and is hyposmic, his movement disability is currently at Hoehn and Yahr stage 3, with an MDS-UPDRS (Movement Disorder Society—Unified Parkinson’s Disease Rating Scale) score (OFF medication) of 39 and a 90% independent Schwab and England assessment. He does not suffer from autonomic dysfunction (assessed by SCOPA-AUT (Scales for Outcomes in Parkinson’s Disease—Autonomic Dysfunction)), daytime sleepiness (Epworth sleepiness scale = 0), or depression (Geriatric Depression Scale score = 30).

The proband’s brother, Tun_IV-7, also presented at age 10 with paroxysmal generalized weakness leading to frequent falls, with diurnal fluctuations and sleep benefit, and tremor in both hands. He has been treated with L-dopa since the age of 19 years old, with a dramatic improvement, without dyskinesias or motor fluctuations. He has no dysphagia, dysarthria, autonomic symptoms, sleep disorders, cognitive impairment, or psychiatric symptoms. His last examination, at age 58, off medication, showed he had mild bradykinesia but no rigidity, with postural tremor and mid dystonia in both hands. He is currently on Madopar (a levodopa/benserazide medication) (375 mg/day) and trihexyphenidyl (7.5 mg/day), similar to his affected brother. Off medication, his assessment is at Hoehn and Yahr stage 2 and 100% independent on the Schwab and England scale, with an MDS-UPDRS score of 19. In both affected brothers, recent MRI scans performed in the past year were unremarkable.

### 2.2. Genomic and Protein Structure Analyses

Exome sequencing for the proband (Pal_IV-3) and both brothers (Tun_IV-3 and Tun_IV-7) revealed homozygosity for *SPR* c.560A>G (Glu187Gly) (NCBI NM_003124.5; dbSNP rs1670577442). In both Tun_IV-3 and Tun_IV-7, optical genome mapping detected a loss of heterozygosity (LOH) in the region chr2:57,805,052–87,431,530 encompassing SPR (chr2:72,887,382–72,892,158). Subsequent Sanger sequencing of *SPR* exon 2 in both families confirmed homozygosity for this missense variant and segregation with disease (Figure 1d). This mutant allele exhibits extreme rarity, as evidenced by its low frequency in public databases: NCBI ALFA G = 0.00000 (0/10,680) and gnomAD V4.1.0 = 6.26 × 10^−7^ (1/1,597,086 alleles). Notably, genetic evaluation excluded a differential diagnosis of alternate conditions, including spinal muscular atrophy (SMA), through negative *SMN1/SMN2* deletion/duplication analysis, and Pelizaeus-Merzbacher disease, as *PLP1* mutation analysis was negative. In Family 2 (Tun_IV-3 and Tun_IV-7) this included exon sequencing and deletion/duplication analysis of *PRKN*, *PINK1* and *DJ-1* as genes for early-onset PD.

**Figure 2:**
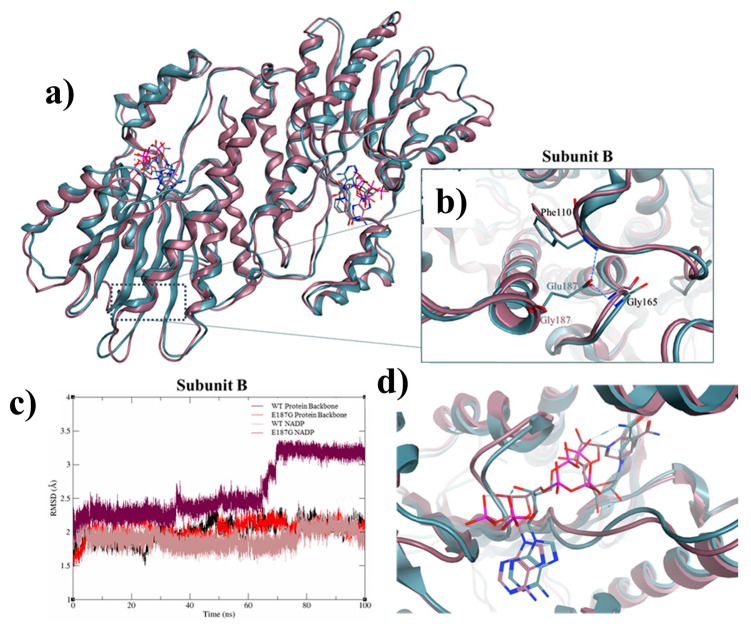
Molecular dynamics simulation analysis of the p.Glu187Gly mutated SR enzyme (**a**) Molecular dynamic simulations highlight the top-clustered conformations of the sepiapterin reductase-NADP complex (from n = 100), with both wild-type (teal ribbon) and mutated structures (pink ribbon) aligned (**b**) Close-up of the Glu187Gly substitution and the disrupted interaction with Phe110 and Gly165 (hydrogen bonding represented as a blue dotted line). (**c**) RMSD analysis of the protein backbone for subunit B, along with the co-substrate NADP, indicates that the mutation has no significant effect on the enzyme’s overall structure. Both the wild-type and mutant forms exhibit relatively low RMSD values, ranging from 1.8 to 2.2 Å throughout most simulations. However, NADP(H)-protein binding interactions display significant instability, particularly after 65 ns, spiking to an average of 3.25 Å. As a result, several interactions, such as Arg40-Asp67-Gly69, Arg435-Ala429, and Ser261-Leu362-Met462 appear disrupted. (**d**) NADP binding in the mutated isoform (pink sticks) appears strained around its benzene ring compared to the extended conformation of the wild-type isoform (blue sticks).

The SR Glu187Gly amino acid substitution is at a species conserved site, and multiple in silico prediction tools predict its deleterious effect on protein structure and enzyme function [7,8,9] (Figure 1e, Table 2). The crystal structure of sepiapterin reductase suggests it exists as a homodimer comprising two subunits, bound to the co-substrate NADP(H) [3]. Modelling shows the Glu187Gly substitution will affect interactions among residues Arg40-Asp67-Gly69, Arg435-Ala429, and Ser261-Leu362-Met462, and it is predicted to disrupt the overall structure of SR and destabilize co-substrate binding and enzymatic function (Figure 2).

## 3. Discussion and Conclusions

Herein, we report the clinical evaluation, diagnostic journey, molecular assessment, and treatment outcomes of three patients in two families with sepiapterin reductase deficiency. Clinical manifestations in the families demonstrate the clinical heterogeneity of SRD, influenced by their age at evaluation. Notably, the patient from the Palestinian family showed early developmental delays with prominent ataxia, hypotonia, and fatigue. In contrast, the Tunisian siblings were mainly affected by juvenile-onset parkinsonism and cognitive impairment. This highlights distinct phenotypic spectra, with a more severe phenotype observed in the younger patient, while parkinsonism predominates in the older cases. Diurnal fluctuation, a characteristic feature of dopa-responsive disorders, was present in both families, but varied in severity. Despite the phenotypic variability, all patients responded positively to L-dopa therapy, which highlights the critical importance of early pharmacologic intervention. Differences in neurological and cognitive profiles might also reflect the compounded impact of delayed diagnosis and treatment initiation, as the Palestinian patient began therapy significantly earlier in life than the Tunisian brothers. Genetic analysis revealed a homozygous mutation in *SPR* c.560A>G (Glu187Gly) that segregates with disease. Subsequent in silico, molecular, and biochemical assessments demonstrated the deleterious effect of this variant on enzyme function, consistent with cerebrospinal fluid analysis of metabolites and neurotransmitters, supporting its pathogenicity.

Establishing a diagnosis required a multidisciplinary approach, considering clinical findings, genetic results, and biochemical assessments [11,12,13]. SRD is classified as a dopa-responsive dystonia syndrome (DYT), along with mutations in *GCH1* and *TH* genes, due to their common features and excellent response to low-dose L-dopa therapy [14,15]. Nevertheless, dystonia is not always present in SRD patients, in whom cognitive and behavioral abnormalities are more frequently observed [16]. All three DYT syndromes show abnormally low CSF levels of 5-hydroxyindoleacetic acid and homovanillic acid, although a differential diagnosis might be made based on the relative concentrations of biopetrin (BP), neopetrin (NP), and sepiapterin (SP) metabolites [5,14]. The biomarker profile of SRD patients shows elevated SP and BP levels with normal NP levels. DYT-GCH1 (MIM: 128230) is characterized by low BP and NP levels with normal SP. An abnormal plasma phenylalanine loading test in patients with dystonia supports a diagnosis of DYT-GCH1, whereas similar biomarkers are normal in patients with DYT-TH (MIM: 605407). However, genetic results most readily and precisely distinguish between these disorders.

The longitudinal progression of patients reported in this study adds to prior clinical observations in a cohort of 43 patients with SRD [6]. Their age of disease onset ranged from birth to six years, with an average diagnostic delay of 9.1 years. Notably, 19 of these patients were misdiagnosed with cerebral palsy (CP). Disease presentations ranged from severe motor and cognitive impairments to patients with mild or minimal symptoms, only identified by family members. Approximately half (45–65%) had motor and speech delays, axial hypotonia, weakness, sleep disturbance, parkinsonian features, and behavioral abnormalities. Notably, less than half had dystonia (40%), which is more prevalent in tetrahydrobiopterin (BH4) disorders. Dopa-responsive dystonia may be absent in patients with early-onset SRD [6] and may not manifest later due to the preventative effect of medication. Diurnal fluctuation is consistently observed in SRD patients, with a prevalence of 75% [6], and was noted in both the Palestinian proband and the two Tunisian patients. In addition, approximately half experience childhood-onset sleep disturbance, cognitive disabilities, and behavioral abnormalities [6]. Given this broad variability, an SRD diagnosis generally requires *SPR* genetic analysis, for which 16 pathogenic, mostly missense, variants have been described (including the current report). Further diagnostic confirmation can be obtained through CSF analysis or impaired enzymatic activity.

Although our patients have experienced symptoms since infancy, a definitive diagnosis was not made until later in their lives; Pal_IV-3 started showing symptoms at a few months of age but was only diagnosed with SRD at 9 years of age. Patients in the Tunisian family only received a genetic diagnosis as adults. Genome-wide testing is now readily available to expedite earlier diagnosis and intervention. All patients show significant improvement in motor function on L-Dopa medication, but the earlier they receive medication, the greater the improvement in motor and cognitive outcomes [17]. Pal_IV-3 has shown improved alertness and sleep after medication, albeit with dose-related dyskinesias and abdominal pain. Increasing the carbidopa dosage along with L-dopa and 5-HTP may modestly relieve treatment-related side effects, as previously reported [6].

In summary, the clinical and molecular investigation of SRD patients provides novel insights into the etiology and ontology of the disease. We highlight the importance of early diagnosis and immediate management, given the potential for dramatic, long-term clinical improvement. Comparing our patients’ clinical phenotypes, diagnostic, and treatment journeys with a broader cohort contributes to a better understanding of the disease spectrum and its therapeutic outcomes. Genetic screening for SRD is recommended in pediatric patients presenting with developmental delay, paroxysmal weakness and fatigue, hypotonia, ataxia, and frequent falls with diurnal fluctuation. In contrast to invasive CSF profiling, genetic testing may simplify and expedite diagnosis and early intervention.

## 4. Methods

### 4.1. Ethics

All participants over the age of 18 years, or their proxies, provided written informed consent prior to study participation, and all procedures were independently reviewed by local research ethics boards. The study in the United Arab Emirates was approved by the Abu Dhabi Health Research and Technology Committee, reference number DOH/CVDC/2021/1318, per national regulations. The father of the Palestinian patient provided written informed consent of participation. A study at the Mongi Ben Hmida National Institute of Neurology in Tunisia was approved by the Ministry of Health. The affected proband and family members provided written informed consent. The Centre for Applied Neurogenetics (CAN) at the University of British Columbia performed exome sequencing and subsequent genotype analysis for the Tunisian family, with appropriate REB approvals (H10-02191, H10-01461, and H11-02030; PI Dr. Matt Farrer). Additional clinicogenetic analyses were performed at the University of Florida (IRB#202000632; #202001661; PI Dr. Matt Farrer).

### 4.2. Clinical and Genealogic Assessment

Longitudinal clinical assessments, including a genealogic history, were obtained for each affected individual and their families. Each proband/family was identified independently at the Metabolic Genetics and Pediatric Neurology Clinic at Tawam Hospital, Abu Dhabi, United Arab Emirates, or at the Mongi Ben Hmida National Institute of Neurology, Tunisia. Standardized measures included neurologic exams, as stated. Tunisian patients were clinically assessed using the following scales: MDS-UPDRS, Hoehn and Yahr, Schwab and England, SCOPA-AUT, MMSE (Mini-Mental State Examination), FAB (Frontal Assessment Battery), ESS (Epworth Sleepiness Scale) and GDS (Geriatric Depression Scale). All family members were asked to provide whole blood samples to enable genetic testing and segregation analysis.

### 4.3. Genomic and In Silico Analyses

Genomic DNA (gDNA) was extracted from blood using a QIAmp Mini Kit (Qiagen, Germany). Affected patients underwent exome sequencing at the UAEU Genomics Laboratory (UAE) or at CAN, University of British Columbia, using TruSeq DNA Exome Library Preparation (Illumina Inc., San Diego, CA, USA) and paired-end sequencing (NovaSeq 6000, NextSeq 500 platforms; Illumina Inc., San Diego, CA, USA) [18]. Bionano optical genome mapping (OGM) was also performed on two affected individuals from the Tunisian family at the Institute of Neurogenetics, University of Lübeck, Lübeck, Germany. Putative pathogenic variants were independently shortlisted in each family according to ACMG criteria [19] to best explain patient phenotypes and patterns of disease inheritance. The most parsimonious variant assignments were confirmed by Sanger sequencing. Notably, genomic primers (forward: 5′-TGAACTTGACCTCCATGCTCTG-3′ and reverse: 5′-TGATGGGTCAAGATTCAAGGAC-3′) were used to amplify a 350 bp product including exon 2 of the *SPR* gene. PCR cycling followed standard conditions for Taq DNA Polymerase (Qiagen Gmbh), and products were purified with ExoSAP-IT (USB Inc., Douglasville, GA, USA) prior to Sanger sequencing using the BigDye Terminator v3.1 kit (Applied Biosystems™, Waltham, MA, USA) on a SeqStudio Genetic Analyzer (Applied Biosystems™, USA). Sequencing data were analyzed using BioEdit Sequence Alignment Editor v7.2.5 (Ibis Biosciences, Carlsbad, CA, USA). Protein Data Bank (PDB) structures were subjected to molecular dynamic (MD) simulations using the AMBER18 package, as previously described [18,20]. Amino acid substitutions were incorporated using MOE software [21] to model the most representative structures for wild-type and mutant protein isoforms.

## Figures and Tables

**Table 1:** CSF neurotransmitter measures in patient Pal_IV-3.

	Patient Result (nmol/L)	Reference Range (nmol/L)
**5-Hydroxyindoleacetic Acid**	7 ↓	66–338
**Homovanillic Acid**	93 ↓	218–582
**3-O-methyldopa**	6	<100
**Sepiapterin**	3.8 ↑	<2.0
**Dihydrobiopterin**	31.4 ↑	3.0–18.0

↓ decreased and ↑ elevated levels.

**Table 2:** In silico prediction analysis of the SPR substitution on enzyme structure and function.

		c.560A>G (Glu187Gly)
**Position**	**Gene**	Exon 2 (Ch2:73115698)
	**Protein**	The last amino acid of the 9th helix
**Allele frequency**		GnomAD V4.1.0 = 6.26 × 10^−7^ G = 0.00000 (0/10,680, ALFA) *
SIFT **	**Effect**	Affect protein function
	**Score**	0.00
**PolyPhen2 ^#^**	**Effect**	Probably damaging
	**Score**	1.0
**PremPS**	**ΔΔG ^†^** **(kcal mol-1)**	0.66
	**Location**	Core
**Conservation**		Highly conserved
**The Hope Project**		The variant is predicted to disturb the enzyme’s overall structure and conformation.
**Pathogenicity** **classification ^††^**		Pathogenic (PP3, PS4, PM2, PP5)

* dbSNP database (rs1670577442). ** The score ranges from 0 to 1. The amino acid substitution is predicted to be damaging if the score is <0.05 and tolerated if the score is >0.05. ^#^ The score ranges from 0.0 (tolerated) to 1.0 (deleterious). ^†^ ΔΔGwt → mut (positive and negative signs correspond to destabilizing and stabilizing mutations, respectively). ^††^ As per the ACMG/AMP guidelines using Franklin by genoox [10].

## Data Availability

Variant data have been submitted to ClinVar with the following submission ID: SUB13929340 for the missense (c.560A>G) variant. All data supporting the study are available on request from the corresponding authors.

## Abbreviations

The following abbreviations are used in this manuscript:

SRD	Sepiapterin Reductase Deficiency
BH4	Tetrahydrobiopterin
HGMD	Human Gene Mutation Database
SR	Sepiapterin Reductase
CSF	Cerebrospinal Fluid
MD	Molecular Dynamics
PDB	Protein Data Bank
DTR	Deep Tendon Reflexes
MMSE	Mini-Mental State Examination
FAB	Frontal Assessment Battery
MDS-UPDRS	Movement Disorder Society—Unified Parkinson’s Disease Rating Scale
SCOPA-AUT	Scales for Outcomes in Parkinson’s Disease—Autonomic Dysfunction
MRI	Magnetic Resonance Imaging
T2WI	T2-Weighted Imaging
NCS	Nerve Conduction Study
EMG	Electromyography
5-HTP	5-Hydroxytryptophan
SMA	Spinal Muscular Atrophy
DYT	Dopa-Responsive Dystonia
BP	Biopterin
NP	Neopterin
SP	Sepiapterin
CP	Cerebral Palsy
